# Optimism and Mental Health in Adolescence: a Prospective Validation Study of the Dutch Life-Orientation Test-Revised (LOT-R-A) for Adolescents

**DOI:** 10.5334/pb.799

**Published:** 2021-03-18

**Authors:** Anne Kennes, Sanne Peeters, Mayke Janssens, Jennifer Reijnders, Marianne Simons, Johan Lataster, Nele Jacobs

**Affiliations:** 1 Faculty of Psychology, Open University, Heerlen, The Netherlands; 2 Department of Psychiatry and Psychology, School for Mental Health and Neuroscience, Maastricht University Medical Centre, Maastricht, The Netherlands

**Keywords:** psychometric properties, positive mental health, well-being, adolescence, optimism

## Abstract

The study aimed to examine the psychometric properties of the Dutch Life Orientation Test-Revised for Adolescents (LOT-R-A), a self-report questionnaire assessing dispositional optimism, and to evaluate the two-factor structure (optimism, pessimism). The LOT-R-A and the questionnaires measuring well-being (MHC-SF-A) and psychological problems (SDQ) were completed by 459 Dutch adolescents (178 boys and 281 girls) between the ages of 11 and 18 years at baseline and 281 adolescents at a four-week follow-up. The results confirmed the two-factor structure (optimism, pessimism) of the LOT-R-A. The findings provided evidence of internal consistency of scores ranging from low to good, and evidence of good test-retest reliability of scores. Further, scores of optimism were cross-sectionally positively associated with scores of positive emotions and well-being and negatively with scores of psychological problems and negative emotions, providing evidence of convergent and divergent validity of optimism scores with emotions, well-being, and psychological problems. Lastly, scores of optimism were prospectively positively associated with scores of well-being and negatively with scores of psychological problems, providing evidence of criterion validity of optimism scores with well-being and psychological problems. Based on these findings it can be concluded that the LOT-R-A is a valid instrument to examine optimism among adolescents. Future research can help to elucidate the role of optimism in mental health interventions and can gather knowledge on how these interventions can be refined to optimally cultivate optimism during the developmental period of adolescence.

Adolescence is characterized by various significant changes in the physical, cognitive, emotional, and social domains (Sisk & Zehr, 2005). Adolescents encounter various developmental challenges and need to acquire the corresponding skills to cope with these (Jackson & Goossens, 2020). Important challenge adolescents have to face is the consolidation of their identity (Jackson & Goossens, 2020). Along with this challenge comes the building of autonomy towards their parents (Brown, 2004; Keyes, 1998). Meanwhile, during this period, adolescents may experience an increase in emotional reactivity and negative emotions (Hampel & Petermann, 2006; Nelson, Leibenluft, Tone, & Pine, 2005). How well adolescents cope with and overcome these developmental challenges, has a significant and long-lasting impact on their mental health (McGorry & van Os, 2013). Therefore, it is important to foster protective factors that facilitate adolescents to deal with these developmental challenges. Optimism, defined as the extent to which individuals hold generalized expectancies of their future outcomes to be beneficial (Carver & Scheier, 1999), and also referred to as dispositional optimism, may be an important protective factor in this context. Indeed, previous research already highlighted the association of optimism with measurements of mental health and showed that optimism predicts resilience in adult samples (Ames, Rawana, Gentile, & Morgan, 2015; Rawana & Ames, 2012; Segovia, Moore, Linnville, Hoyt, & Hain, 2012; Monzani, Steca, & Grec, 2014; Smokowski, Evans, Cotter, & Webber, 2014; Vacek, Coyle, & Vera, 2010).

To further extend our knowledge on the role of optimism in fostering adolescent’s mental health, a reliable and valid instrument to assess levels of optimism among adolescents is needed. The most commonly used instrument to measure optimism is the Life Orientation Test–Revised (LOT-R; Scheier, Carver & Bridges, 1994). Therefore, this study adapted the Dutch adult version of the LOT-R (ten Klooster et al., 2010) to a target group of Dutch adolescents by rephrasing incomprehensible and difficult words to improve understandability. Further, this study investigated the factor structure and the psychometric properties of the adapted questionnaire (hereafter referred to as LOT-R-A). Previously, the Life-Orientation Test-revised (LOT-R) has been validated in adult samples from the Netherlands, Germany, Greece, Spain, America and Latin America (Cano-García et al., 2015; Hinz et al., 2017; ten Klooster et al., 2010; Lyrakos et al., 2010; Scheier, et al., 1994; Zenger et al., 2013) and in adolescent samples from Italy, Japan, and Siberia (Jovanovic & Gavrilov-Jerkovic, 2013; Monzani et al. 2014; Sumi, 2004). This paper first examines the factor structure of the LOT-R-A, followed by assessing the internal reliability and test-retest reliability of scores. Secondly, the paper investigates the convergent and divergent construct validity of optimism scores with emotions, well-being and psychological problems by examining cross-sectional associations between scores of optimism and positive and negative emotions, well-being, and psychological problems. Lastly, the prospective association between optimism and mental health is included as evidence of criterion validity of optimism scores with well-being and psychological problems, complementing previous validation studies of the LOT-R in adolescent samples (Jovanovic & Gavrilov-Jerkovic, 2013; Monzani et al. 2014; Sumi, 2004).

Concerning the factor structure of the LOT-R, controversy had arisen on how optimism and pessimism relate to each other. Initially, optimism was regarded as a single bipolar dimension with optimism and pessimism as opposite ends of the same dimension. Although the LOT-R was developed to measure optimism (i.e., calculated as the sum of the three positively formulated items and the three inverted negatively formulated items) as a one-dimensional concept (Scheier et al., 1994), factor analytic studies, predominantly in adult samples, have suggested a two-factor structure (optimism; pessimism) with one factor representing optimism (i.e., calculated as the sum of the three positively formulated items) and the other representing pessimism (i.e., calculated as the sum of the three negatively formulated items) (Glaesmer et al., 2012, Gustems-Carnicer, Calderón, & Santacan, 2017; Herzberg, Glaesmer, & Hoyer, 2006, Hinz et al., 2017, ten Klooster et al., 2010). Contrary to the one-dimensional conceptualization of optimism, this structure considers optimism and pessimism as two different and distinct dimensions. In both adult and adolescent samples, the majority of the LOT-R validation studies found evidence for a two-factor structure (optimism, pessimism) (Hinz et al., 2017; ten Klooster et al., 2010; Lyrakos et al., 2010; Scheier et al., 1994; Zenger et al., 2013; Jovanovic & Gavrilov-Jerkovic, 2013; Sumi, 2004). Pessimism cannot be regarded as a lack of optimism. Other studies demonstrated that the single bipolar dimension of optimism could be preserved by introducing an extra dimension, a response style (i.e., calculated as the sum of the three positively formulated items) related to impression management (Alessandri et al., 2010; Cariou, Raufaste, & Vautier, 2003; Monzani et al., 2014; Rauch, Schweizer, & Moosbrugger, 2007). In sum, three different dimensional models are observed in previous studies including adults and adolescents: a one-factor model, a two-factor model consisting of optimism and pessimism, and a two-factor model consisting of optimism and response style. As the findings confirmed invariance of the two-factor model across age groups (Hinz et al., 2017) and a previous validation study of the LOT-R in a Dutch adult sample (ten Klooster et al., 2010) observed the two-factor structure (optimism, pessimism), it is expected to confirm the two-factor structure (optimism, pessimism). Further, it is expected that the findings provide evidence of high internal reliability of scores and evidence of good test-retest reliability of scores, similar to previous studies (Glaesmer et al., 2012; Gustems-Carnicer et al. 2017; Hirsch, Britton, & Conner, 2010; Scheier et al., 1994).

Additionally, construct validity of optimism scores with emotions, well-being and psychological problems is investigated with the cross-sectional associations between optimism scores and scores of positive and negative emotions, psychological problems, and well-being. Previous research has shown that optimistic beliefs are not only associated with higher levels of positive emotions but also with lower levels of negative emotions (Murberg, 2012; Puskar, Sereika, Lamb, Tusaie-Mumford, & McGuinness, 1999). Further, in adolescent samples, higher levels of optimism have been associated with lower levels of psychological problems, such as mood and anxiety disorders (Ames et al., 2015), externalizing behaviors (Smokowski et al., 2014), and substance use (Rawana & Ames, 2012). Therefore, it is expected that scores of optimism are positively associated with scores of positive emotions and negatively associated with scores of psychological problems and negative emotions. Furthermore, it is expected that optimism is also associated with well-being. Indeed, previous research has shown that optimism is positively associated with EWB (Monzani et al., 2014; Vacek et al., 2010). In addition to EWB, well-being can also be defined as optimal psychological functioning both on a personal and social level, conceptualized as, respectively, psychological well-being (PWB) and social-well-being (SWB; Keyes, 2002). In contrast with EWB, less research is available studying the associations between dispositional optimism and these aspects of well-being in an adolescent sample. However, a number of mechanisms may link optimism positively to well-being and negatively to psychological problems. The expectancy-incentive model of motivation assumes that the individuals’ motivation to fit their behavior in order to attain a desired goal, depends on the expectancy i.e. confidence that the goal can be obtained (Carver, 2014). Compared to less optimistic adolescents, optimistic adolescents have more confidence in their future, and stronger believes that they will achieve their goals. When setbacks occur, they use more active forms of coping and exhibit behavior that leads to comparatively more success (Andersson, 1996; Carver & Scheier, 1999). Successfully achieving goals might lead to personal growth, a facet of PWB, and satisfaction, whereas problems in overcoming setbacks may cause psychological problems (Erikson, 1968; McGorry & van Os, 2013). However, adolescents cannot always attain their desired goals. This may explain why, besides tenacious goal pursuit, flexible goal adjustment has been found to be an even more important underlying mechanism of the link between optimism and mental health (Hanssen et al., 2015, Wrosch & Scheier, 2003). Optimism is linked with goal disengagement and re-engagement (Hanssen et al., 2015). Compared to optimistic adolescents, less optimistic adolescents have less confidence in their future outcomes and are less inclined to disengage their goals (Carver, Schreier, & Segertrom, 2010). Flexible goal adjustment protects adolescents from pursuing unattainable goals and the corresponding decrease in EWB and increase in psychological problems (Wrosch & Scheier, 2003). Flexible goal adjustment might additionally underlie the link between optimism and PWB. Disengagement of unattainable goals frees resources (e.g., time) that can be used for other, more attainable goals, thereby enabling successful personal growth, a facet of PWB (Keyes, 1998). A third mechanism that may underlie the link between optimism and mental health is the superior adjustment to stressful life events exhibited by optimistic adolescents (Brissette, Scheier, & Carver, 2002). Optimists cope more effectively with stressors (Brissette et al., 2002), as they switch more flexibly between different coping strategies, in order to find the most effective strategy to deal with the situation (Carver et al., 2010; Solberg, Nes, & Segerstrom, 2006). On top of that, optimists use more active forms of coping and fewer avoidance strategies (Scheier & Carver, 1985). Active coping strategies are linked with higher levels of EWB and resilience in response to a variety of stressors (Coyle & Vera, 2013), whereas the use of avoidance strategies is linked to psychological problems (Arslan, 2017). Furthermore, optimists tend to have more positive and supportive social relationships than pessimists and possess a more extensive social network, regarding both close and distant relationships (Brissette et al., 2002, Carver et al., 2010). Research has shown that people, in general, tend to accept and to like optimists more easily than less optimistic people (Carver et al., 2010). Optimists also have a more positive perception of their social relationships: they feel more accepted, are more satisfied with their relationships, and are also prepared to work harder to maintain them (Carver et al., 2010). Thus, taken together, a number of mechanisms seem to link optimism positively to well-being across all aspects of well-being (EWB, PWB, and SWB). Therefore, it is expected that scores of optimism are positively cross-sectionally associated with scores of well-being.

Previous longitudinal studies of optimism in adolescent samples also provided evidence for a prospective association between optimism and mental health. In particular, it has been suggested that optimism can be considered as a predictor of psychological problems (e.g., depression, anxiety) (Kleiman et al., 2017) as well as a predictor of well-being (Phan, 2016, Rand, Shanahan, Fischer, & Fortney, 2020; Sulkers et al., 2013). Therefore, in order to confirm the criterion validity of optimism scores with well-being and psychological problems, it is expected that optimism scores at baseline are positively associated with scores of well-being at follow-up and negatively associated with scores of psychological problems at follow-up.

To summarize, this research investigates the factor structure and the psychometric properties of the LOT-R-A. It is expected that the two-factor structure (optimism, pessimism) is confirmed in a Dutch adolescent sample. Additionally, it is hypothesized that the findings provide evidence of good test-retest reliability of scores. Furthermore, it is expected that scores of optimism are cross-sectionally positively associated with scores of positive emotions and well-being and negatively with scores of psychological problems and negative emotions, confirming convergent and divergent validity of optimism scores with emotions, well-being and psychological problems. Lastly, it is hypothesized that baseline scores of optimism are prospectively positively associated with scores of well-being at follow-up and negatively with scores of psychological problems at follow up, confirming criterion validity of optimism scores with well-being and psychological problems.

## Method

### Participants

A sample of 459 Dutch adolescents between the ages of 11 and 18 years participated in the current study. Of this initial sample, 281 participants (61.7%; hereafter referred to as completers) completed the survey at follow-up. Compared to participants who only completed the baseline survey (hereafter referred to as drop-outs), the completers had significantly higher education levels, were slightly younger, and reported a higher score on optimism and a lower score on pessimism (***Table 1***).

**Table 1:** Sample characteristics dropouts and completers and differences between dropouts and completers at baseline.


	DROPOUTS (N = 176)	COMPLETERS (N = 281)	COMPLETERS VERSUS DROPOUTS

Age M(SD)	15.34 (1.90)	14.32 (1.97)	t(455) = –5.32*

*Gender*			

Boys (%)	39.8	38.1	χ^2^ (1) = .123

Girls (%)	60.2	61.9	

*Country of birth*			

Born in the Netherlands (%)	93.2	95.4	χ^2^ (1) = 1.265

Born abroad (%)	6.8	4.6	

*Living-situation*			

Living with both parents (%)	75.6	77.2	χ^2^ (1) = .201

Others (%)	24.4	22.8	

*Education level*			

High^1^ (%)	38.1	54.4	χ^2^ (1) = 11.228*

Low^2^ (%)	61.9	45.6	

Optimism (3 items) M(SD)	2.52 (.74)	2.69 (.67)	t(455) = 2.56*

Pessimism (3 items) M(SD)	1.86 (.81)	1.64 (.83)	t(455) = –2.85*


*Note*: * *p* < .05; 1: higher than vocational education 2: vocational education; M = Mean; SD = standard deviation.

### The Life Orientation Test–Revised form for Adolescents (LOT-R-A)

The original LOT-R (Scheier et al., 1994) is a self-report questionnaire, measuring dispositional optimism. The questionnaire consists of 10 items, of which three positively formulated items (1, 4, and 10), three negatively formulated items (3, 7, and 9), and four “filler” items (2, 5, 6, and 8). Respondents indicated the extent to which they agreed with each item on a 5-point Likert scale ranging from strongly agree to strongly disagree. The optimism score was calculated as the sum of the three positively formulated items. The pessimism score was calculated as the sum of the three negatively formulated items.

An example of an item is: “I rarely count on good things happening to me”. Internal reliability of the adult version is acceptable, with a Cronbach’s Alpha of 0.82 (Scheier et al., 1994). For this study, the validated Dutch adult version of the LOT-R (ten Klooster et al., 2010), was adapted by a psychologist to a target group of Dutch adolescents, by rephrasing incomprehensible and difficult words to improve understandability. The psychometric properties of the LOT-R-A are discussed in the results section.

### Mental Health Continuum-Short Form for Adolescents (MHC-SF-A)

The MHC-SF-A (Kennes et al., 2020) is a validated Dutch adolescent version of the MHC-SF (Keyes et al., 2008), a self-report questionnaire, measuring emotional, psychological, and social components of well-being. This questionnaire consists of 14 items scored on a 6-point Likert scale to assess the frequency of every feeling in the past month. An example of an item is: “During the past month, how often did you feel happy?”. Items belonging to the same subscale are added together to generate sum scores for respectively EWB, PWB, and SWB. The psychometric properties of the MHC-SF-A are acceptable, with moderate to high internal consistency (Cronbach’s α of .70 to .84 for different subscales) and moderate test-retest reliability (correlations of .45 to .53) (Kennes et al., 2020).

### Kidscreen-52

Kidscreen-52 (Ravens-Sieberer et al., 2008) is a self-report questionnaire for children and adolescents, measuring ten different dimensions of subjective perceptions of health and well-being: physical well-being, psychological well-being, moods and emotions, self-perception, autonomy; relations with parents and home life, social support and peers, school environment, social acceptance, and financial resources. The dimension “psychological well-being” (hereafter referred to as positive emotions) includes items covering positive emotions. The dimension “moods and emotions” (hereafter referred to as negative emotions) includes items measuring negative emotions, and stressful feelings. The 52 items were scored on a 5-point Likert scale to assess the frequency (never-seldom-sometimes-often-always) of attitudes or certain behaviors/feelings. Items belonging to one subscale were added together to generate a sum score for that subscale. An example of an item is: “Remember the past week. Have you had fun?”. Previous studies have shown that the test-retest reliability of the questionnaire varies between .56 to .77, and Cronbach’s α (internal consistency) ranges from .76 to .89 (Ravens-Sieberer at al., 2008).

### The Strengths and Difficulties Questionnaire (SDQ)

The Dutch self-report version of the SDQ (Muris, Meesters, & Van Den Berg, 2003) was used to measure the behavioral and emotional problems of adolescents. The SDQ consists of five subscales, with four subscales focusing on difficulties relating to behavior, emotional functioning, hyperactivity/inattention, interaction with peers, and one subscale focusing on the strength of prosocial behavior. Each subscale focusing on difficulties consists of five items, indicating the magnitude of problems relating to that difficulty. An example of an item is: “I worry a lot”. The items were scored on the basis of a 3-point scale (not true = 0, a little true = 1, certainly true = 2). A total difficulty score was obtained by calculating the sum of all items of the four scales focusing on difficulties (Muris et al., 2003). This score indicates to what extent the adolescent suffered from socio-emotional problems. The internal consistency of the SDQ is acceptable for both the total difficulty score (Cronbach’s α of 0.70) and for the subscales (Cronbach’s α between .60 and .65) (Muris et al., 2003). Muris et al. (2003) found satisfactory test-retest reliability of the various subscales. All subscales, except the prosocial behavior subscale (.59), had an interclass correlation coefficient of .70 or more (Muris et al., 2003).

### Procedure

Dutch speaking adolescents between the ages of 11 and 18 years were primarily recruited, by undergraduate psychology students, through six secondary schools spread over the Netherlands. Besides being Dutch-speaking, there were no in– and exclusion criteria for participation. Participants were invited to take part in a two-wave online study, measuring optimism, well-being, and psychological problems both at baseline and at follow-up four weeks later. The study was approved by the local research ethics committee of Open University and was carried out in accordance with the Code of Ethics of the World Medical Association (Declaration of Helsinki) for medical research involving humans (World Medical Association, 2013). At the start of the study, written informed consent was obtained from adolescent participants. Parental consent was obtained from adolescents younger than 16 years old. In the informed consent, ethical and privacy issues were covered. Confidentiality as well as anonymity were ensured.

### Data Analysis

The data was analyzed using SPSS 25.0 and R 3.4.3 (R Core Team, 2019). Prior to analyses, negatively phrased items of the LOT-R-A were inverted. Data for all variables was inspected for missing values. If only one item was missing on a scale, the score was summed and divided by the number of items completed. If more items were missing, data from the participant was excluded from analysis. Descriptive statistics, means, and standard deviations were calculated for the main study variables. Confirmatory factor analyses (CFA) were performed to test the factor structure of the LOT-R-A. Three conceptual models were evaluated: a single factor model fitting optimism as the summation of the six items, a two-factor model (optimism and pessimism) with one factor representing optimism as the summation of the three positive items and the other factor representing pessimism as the summation of the three negative items and a two-factor model (optimism and response style) with one factor representing optimism as the summation of the six items and the other factor representing a response style as the summation of the three positive items. Maximum likelihood was used as estimation method. To assess the goodness of fit, the chi-square (χ2), comparative-fit-index (CFI), Tucker-Lewis index (TLI), standardized root-mean-square residual (SRMR), Root Mean Square Error of Approximation (RMSEA), Akaike’s information criterion (AIC), and Bayesian information criterion (BIC) were calculated. CFI and TLI values above .90 (Kline, 1998; Tabachnick & Fidell, 2001), χ^2^/*df* below 5 (Arbuckle, 2016), RMSEA below .08 (Browne & Cudeck, 1992), and SRMR values below .08 (Hu & Bentler, 1999) were considered to indicate an adequately parameterized model. Lower AIC and BIC values were considered indicative of better model fit (Akaike, 1974; Stone, 1979). Exploratory factor analyses were additionally conducted to gain insight into potential standardized factor loadings for optimism in the different models. Internal consistency of the subscales was determined by McDonald’s omega (Peters, 2014). The test-retest reliability was evaluated using the intraclass correlation coefficient (ICC) with a two-way random-effects model with absolute agreement (Shrout & Fleiss, 1979). Values over .70 were considered as good (Terwee et al., 2007). Using baseline data, bivariate cross-sectional correlations of optimism and pessimism with positive validation measures (i.e., positive emotions and the MHC-SF-A subscales) and negative validation measures (i.e., negative emotions and psychopathology) were performed to test convergent and divergent validity. Correlations around .20 were considered to be low, and around .50 to be moderately high (Nunnally & Berstein, 1995). To examine the criterion validity of optimism scores with well-being and psychological problems, regression analyses were performed with the MHC-SF-A subscale (at follow-up) as a dependent variable, and baseline scores of optimism and the corresponding MHC-SF-A subscale as independent variables. Additionally, a regression analysis was performed with psychological problems (at follow-up) as a dependent variable, and baseline scores of optimism, and psychological problems as independent variables. All regression analyses were corrected for gender, age, and education level. Multicollinearity was checked.

## Results

### Structural validity

***Table 2*** shows the fit indices for the three structural models of the LOT-R-A. Only the two-factor models fulfilled fit criteria for all indices, indicating adequate model fit, whereas the one-factor model did not meet all fit criteria. In addition, the latent variables optimism and pessimism did not correlate strongly (r = –.32) in the two-factor (optimism and pessimism) structure, lending further support for the conceptualization of optimism and pessimism as two distinct dimensions rather than opposite ends of the same dimension. As the two-factor structure including optimism and pessimism showed adequate fit and models optimism and pessimism as two distinct dimensions, in contrast to the two-factor structure including optimism and response style, this model was preferred. Consequently, in the current study, optimism was operationalized as the summation of three items. ***Table 3*** displays the standardized factor loadings in the two-factor (optimism, pessimism) structure that was supported by CFA. In order to analyze the internal consistency of the model, the correlation between the score of the items and the corresponding scale was calculated. All factor loadings exceeded the minimum acceptable value of .30 (Nunnally & Bernstein, 1995).

**Table 2:** CFA Models of the latent structure of the LOT-R-A.


	ONE-FACTOR	TWO-FACTOR (OPTIMISM, PESSIMISM)	TWO-FACTOR (OPTIMISM, RESPONSE STYLE)

χ^2^ (df)	38.827(9)*	20.391(8)*	13.163(5)*

CFI	.924*	.968*	.979*

TLI	.873	.940*	.937*

RMSEA	.085	.058*	.060*

SRMR	.048*	.034*	.027*

AIC	7588	7572	7571

BIC	7605	7651	7661


*Note*: * Meeting minimally acceptable fit criteria; χ^2^ = Satorra-Bentler scaled chi-square; df = degree of freedom; CFI = comparative fit index; TLI = Tucker-Lewis index; RMSEA = root mean square error of approximation; SRMR = standardized root mean square residual; AIC = Akaike’s information criterion; BIC = Bayesian information criterion.

**Table 3:** Standardized factor loadings for the two-factor (optimism, pessimism) structure.


	TWO-FACTOR(OPTIMISM, PESSIMISM)	

	OPTIMISM	PESSIMISM

Item 1	.373	

Item 4	.518	

Item 10	.661	

Item 3		.553

Item 7		.579

Item 9		.731


### Characteristics of the Main Study Variables

***Table 4*** summarizes the descriptive statistics of the main study variables at baseline.

**Table 4:** Mean, SD of the main study variables at baseline (n = 459).


	M	SD	MIN	MAX

Optimism (LOT-R-A)	2.63	.70	.00	4

Pessimism (LOT-R-A)	1.72	.83	.00	4

EWB (MHC-SF-A)	3.87	.84	.67	5

PWB (MHC-SF-A)	3.64	.84	.83	5

SWB (MHC-SF-A)	3.11	.85	.80	5

Positive emotions (Kidscreen)	24.53	4.03	12	30

Negative emotions (Kidscreen)	11.78	4.95	6	30

Psychological problems (SDQ)	10.36	5.48	0	32


*Note*: M = Mean; SD = standard deviation EWB = emotional well-being; PWB = psychological well-being; SWB = social well-being; MHC-SF-A = Mental Health Continuum-Short form for Adolescents; SDQ = Strengths and Difficulties Questionnaire.

### Reliability

The McDonald’s omega of the scales were .55 (optimism) and .66 (pessimism). Values of the intraclass correlation coefficients were .74 (optimism) and .67 (pessimism).

### Construct Validity

Collinearity statistics indicate that all VIF values were less than 1.5 (maximum ≤ 5 (Field, 2013)), not indicative of multicollinearity in any of the models under investigation. ***Table 5*** shows the bivariate cross-sectional correlations of optimism and pessimism with the validation measures at baseline. All correlations were statistically significant. Optimism correlated positively with positive outcomes and negatively with negative outcomes. Pessimism correlated negatively with positive outcomes and positively with negative outcomes.

**Table 5:** Bivariate cross-sectional correlations of optimism with validation measures at baseline (n = 459).


	OPTIMISM	PESSIMISM

Positive emotions (Kidscreen)	.613*	–.428*

Negative emotions (Kidscreen)	–.580*	.470*

EWB (MHC-SF-A)	.529*	–.354*

PWB (MHC-SF-A)	.574*	–.371*

SWB (MHC-SF-A)	.435*	–.129*

Psychological problems (SDQ)	–.509*	.498*


*Note*: * *p* < .05; EWB = emotional well-being; PWB = psychological well-being; SWB = social well-being; MHC-SF-A = Mental Health Continuum-Short form for Adolescents; SDQ = The Strengths and Difficulties Questionnaire.

### Criterion Validity

***Table 6*** shows the results of the regression analyses. Higher scores of optimism at baseline were prospectively associated with higher scores of EWB, PWB, and SWB at follow-up. In addition, higher scores of optimism at baseline were prospectively associated with lower scores of psychological problems at follow-up.

**Table 6:** Regression analyses predicting well-being and psychological problems at follow-up.


PREDICTOR	EWB B (SE)	PWB B (SE)	SWB B (SE)	PSYCHOLOGICAL PROBLEMS B (SE)

F(4, 280)	72.9*	83.9*	70.2*	71.3*

R^2^	51%	55%	51%	52%

Age	–.096(.021)*	–.066(.020)*	–.094(.021)*	.241(.033)*

Gender	–.089(.080)	–.026(.076)	–.026(.087)	–.042(.126)

Education level	–.013(.078)	–.092(.073)	–.046(.076)	.250(.123)

EWB (baseline)	.493 (.055)*			

PWB (baseline)		.594 (.053)*		

SWB (baseline)			.579 (.050)*	

Psychological problems (baseline)				.121 (.013)*

Optimism	.326 (.070)*	.250 (.068)*	.153 (.064)*	–.261 (.109)*


*Note*: * *p* < .05; EWB = emotional well-being; PWB = psychological well-being; SWB = social well-being; SE = Standard Error.

## Discussion

The paper aimed to investigate the factor structure and the psychometric properties of the Life Orientation Test–Revised form for Adolescents (LOT-R-A). As a valid Dutch instrument to measure optimism was lacking, this study adapted the Dutch adult version of the LOT-R (ten Klooster et al., 2010) to a target group of Dutch adolescents and examined the factor structure of the LOT-R-A. In line with expectations, the two-factor (optimism and pessimism) structure showed an adequate fit to the data, confirming previous studies in both adult and adolescent samples (Hinz et al., 2017; ten Klooster et al., 2010; Lyrakos et al., 2010; Scheier et al., 1994; Zenger et al., 2013; Jovanovic & Gavrilov-Jerkovic, 2013; Sumi, 2004). Although optimism and pessimism tend to correlate more strongly in younger age groups (Herzberg et al., 2006), they can be seen as two different and distinct dimensions in adolescence. These findings indicate that pessimism cannot be simply regarded as a lack of optimism, or in other words, stimulating generalized positive expectations for the future is not the same as preventing generalized negative expectations for the future. In addition to the structure of the LOT-R-A, this study also evaluated its psychometric properties. The estimation of internal consistency of the optimism score was low which may be partly due to the small number of items – only three – measuring optimism (Tavakol & Dennick, 2011). Furthermore, in all the model structures, the factor loading of the first optimism item was low, which may have negatively affected internal consistency. In contrast with validation studies of the LOT-R in adult samples (e.g., Glaesmer et al., 2012, Gustems-Carnicer et al., 2017), validation studies of the LOT-R in adolescent samples (e.g., Monzani et al., 2014) also showed a low factor loading of this item. This might suggest that this item is less suitable for measuring optimism in an adolescent sample. The estimation of test-retest reliability of the optimism score was good. This suggests that scores on the LOT-R-A are stable over time but still sensitive enough to measure changes in optimism. The estimation of test-retest reliability of the pessimism score was moderate. Further, the results provided evidence of convergent and divergent validity of optimism score with emotions, well-being, and psychological problems. In line with our expectations, scores of optimism were cross-sectionally positively associated with scores of positive emotions and well-being and negatively with scores of psychological problems and negative emotions. Lastly, the results provided evidence of criterion validity of optimism scores with well-being and psychological problems. Scores of optimism were prospectively positively associated with scores of well-being and negatively with scores of psychological problems. The LOT-R-A score of optimism is not only an indication of adolescents’ current mental health but might also be indicative for their short-term mental health. Higher levels of optimism appear to be associated with a stronger short-term decrease in psychological problems and a stronger short-term increase in well-being, suggesting that optimism might have the potential to play a role in preserving mental health. However, as the period of time between baseline and follow-up was rather short in this study, caution should be taken when interpreting the longitudinal data. Future research is needed to gain insight into the prospective association of optimism with measurements of mental health on a longer-term. Research has shown that an optimistic view can be learned or improved (Peters, Meevissen, & Hanssen, 2013). Fostering optimism could, for example, be achieved through the Best Possible Self visualization, of which positive effects on optimism have been demonstrated (Peters et al., 2013). Future research can help to elucidate the role of optimism in mental health interventions and therapy and can gather knowledge on how these interventions can be refined to optimally cultivate optimism during this developmental period of adolescence (e.g., Schonert-Reichl & Lawlor, 2010).

In sum, the findings provided evidence of internal consistency of the scores, good test-retest reliability of scores, construct validity of optimism score with emotions, well-being, and psychological problems, and criterion validity of optimism score with emotions, well-being, and psychological problems Based on these findings it can be concluded that the LOT-R-A is a valid instrument to examine optimism among adolescents. As little research is available about pessimism, it is difficult to provide evidence of validity of pessimism score with affect, well-being, and psychological problems. Further, future research should investigate measurement invariance of the LOT-R-A across subgroups of age or gender to determine whether the instrument can be used to reliably compare levels of optimism between adolescents and adults or between subgroups of a different gender.

The current study had several strengths. A large adolescent sample from different regions of the Netherlands was recruited through secondary schools and participants completed the online questionnaire twice, once at baseline and once four-weeks later. Nevertheless, this study had several limitations. Firstly, while the dropout rate at follow-up was considered acceptable (38%), consideration should be given to a possible selectivity in study dropout, as there was a tendency for relatively older, lower educated, and less optimistic participants to more likely drop out of the study. Secondly, to evaluate the psychometric properties of the LOT-R-A, self-report measures were used, which might have led to response bias. Response biases can be minimized by using a multi-modal approach where observations or interviews are used in combination with self-report measures (Paulhus & Vazire, 2007). Thirdly, although efforts were made to collect a representative sample, boys were relatively underrepresented in our study (38.2% vs. 50% at the population level, CBS, 2020). However, compared to the Dutch population (CBS, 2020), the sample was representative in terms of educational level and migration background. This study possibly involved an oversampling of mentally healthy adolescents. Future studies should address whether these findings are also applicable to adolescents with mental health problems.

## Conclusion

The findings provided evidence of good test-retest reliability of scores, construct validity of optimism score with emotions, well-being, and psychological problems, and criterion validity of optimism score with emotions, well-being, and psychological problems, supporting the use of the LOT-R-A in Dutch adolescents to measure optimism. Furthermore, in the short term, optimism was prospectively positively associated with well-being and negatively associated with psychological problems. Future research can help to elucidate the role of optimism in mental health interventions and can gather knowledge on how these interventions can be refined to optimally cultivate optimism during the developmental period of adolescence.
